# Copolymerization of a Catechol and a Diamine as a Versatile Polydopamine-Like Platform for Surface Functionalization: The Case of a Hydrophobic Coating

**DOI:** 10.3390/biomimetics2040022

**Published:** 2017-11-13

**Authors:** Salvio Suárez-García, Josep Sedó, Javier Saiz-Poseu, Daniel Ruiz-Molina

**Affiliations:** Catalan Institute of Nanoscience and Nanotechnology (ICN2), CSIC and BIST, Campus UAB, Bellaterra, 08193 Barcelona, Spain; salvio.suarez@icn2.cat (S.S.-G.); josep.sedo@icn2.cat (J.S.)

**Keywords:** catechol, coating, surface modification, hydrophobicity, cross-linking, polydopamine, surface functionalization

## Abstract

The covalent functionalization of surfaces with molecules capable of providing new properties to the treated substrate, such as hydrophobicity or bioactivity, has been attracting a lot of interest in the last decades. For achieving this goal, the generation of a universally functionalizable primer coating in one-pot reaction and under relatively mild conditions is especially attractive due to its potential versatility and ease of application. The aim of the present work is to obtain such a functionalizable coating by a cross-linking reaction between pyrocatechol and hexamethylenediamine (HDMA) under oxidizing conditions. For demonstrating the efficacy of this approach, different substrates (glass, gold, silicon, and fabric) have been coated and later functionalized with two different alkylated species (1-hexadecanamine and stearoyl chloride). The success of their attachment has been demonstrated by evaluating the hydrophobicity conferred to the surface by contact angle measurements. Interestingly, these results, together with its chemical characterization by means of X-ray photoelectron spectroscopy (XPS) and Fourier-transform infrared spectroscopy (FT-IR), have proven that the reactivity of the primer coating towards the functionalizing agent can be tuned in function of its generation time.

## 1. Introduction

Macro- and micro/nano-surface functionalization by means of thin films with thicknesses measuring a few nanometres has become a topic of considerable interest in the past years due to the possibility of tuning their physico-chemical properties without significantly affecting bulk features or particle sizes. In this sense, the generation of molecular self-assembled monolayers (SAMs) represents a relatively simple and efficient way for surface modification [1]. Nevertheless, this approach usually requires chemical specificity between substrate and anchoring group. For example, thiols are useful for noble metals such as gold [2,3,4,5,6], platinum [7,8,9,10] and palladium [7,11], phosphates and phosphonates for metal oxides [12,13,14,15] and silanes for hydroxyl-bearing materials such as cellulose [16,17] and silicon oxides [18,19,20,21,22], zinc oxides [23,24], iron oxides [25,26], boron [27], and alumina [6] among others. In order to overcome this limitation, several research groups have drawn inspiration from mussel adhesive proteins, since it is known that they are able to strongly stick onto virtually any kind of surface, thus being usable as models for universal anchoring elements [28,29,30]. Research on these proteins suggests that the presence of considerable amounts of the non-essential catecholic amino acid l-3,4-dihydroxyphenylalanine (l-DOPA) is responsible, to a large extent, for this gluing effect, and particularly to the presence of the catechol moiety, as has been demonstrated in single molecule experiments [31]. Thus, catechol-based molecular SAMs could be good candidates for the functionalization of almost any kind of surface. Nonetheless, stable SAMs can only be obtained on certain substrates where the interaction between catechol and surface is strong enough for achieving a proper stabilization of the molecular monolayer. This is the case of some metal oxides, where a coordination bond is established [32,33,34]. If only weak interactions come into play, such as hydrogen bonding, electrostatic forces or π–π stacking, cooperativity between catechol moieties is essential for generating a stable thin film on the surface. This cooperativity can be achieved by polymerizing a catechol bearing a certain functional group whose properties are to be transferred to the treated surface, such as hydrophobicity [35], or making the catechol part of a more complex polymeric backbone [36,37,38,39]. However, this strategy usually entails following more or less complex synthetic pathways for obtaining the final catechol-based coating material. An alternative approach is the self-polymerization of catecholic molecules under oxidative conditions for obtaining coatings with thicknesses of few nanometres, which can be later functionalized. This is the case of polydopamine, which is obtained by oxidative polymerization of dopamine [40]. This first coating, which has been reported to adhere to a wide range of materials [40], can be reacted a posteriori with an alkanethiol, for example, for transferring its hydrophobicity to a given substrate [41]. Based on this same strategy, several authors have been searching for alternative approaches to polydopamine using other catechols as anchoring elements, like caffeic acid [42] and gallic acid [43,44], which are copolymerized along with diamines to favor their cross-linking. It has been demonstrated that the successfully generated copolymer films can be later tailored with different kinds of molecules thanks to the presence of reactive groups like amines and carbonyls.

**Figure 1:**
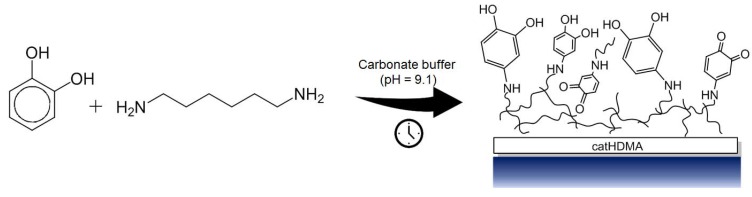
Schematic representation of the copolymerization between pyrocatechol and hexamethylenediamine (catHMDA).

In the present work, we report the copolymerization, under mild basic conditions, of pyrocatechol with different nitrogen-based cross-linkers. As a proof of concept, the obtained polymeric primer is functionalized ex situ by reaction with different alkyl-bearing molecules with the objective of transferring their hydrophobicity to the treated substrates. The chemical and morphological characterization of the coatings at different stages of the process demonstrate that the copolymerization of the simplest catecholic molecule with hexamethylenediamine (HMDA) is enough for successfully generating a coating that can be later used as a chemically functionalizable platform onto a wide range of materials (Figure 1).

## 2. Materials and Methods

### 2.1. Materials

All reagents were purchased and used without further purification from Sigma-Aldrich (Madrid, Spain). Solvents were used as received without additional drying or degasification. Aqueous carbonate buffer (pH = 9.1) was prepared by dissolving 265 mg of Na_2_CO_3_ and 1.89 g of NaHCO_3_ in 250 mL of Milli-Q^®^ water (Merck Chemicals & Life Science S.A., Madrid, Spain). Glass (cut from microscope borosilicate glass slides (Labbox, Madrid, Spain)); and silicon substrates (CNM, Barcelona, Spain) were cleaned in an ultrasonic bath for 10 min, first in acetone and then in ethanol, rinsed with Milli-Q^®^ water and finally dried under a nitrogen flow. Gold-coated glass substrates (Labbox) for Fourier-transform infrared spectroscopy (FT-IR) analysis were obtained by deposition with a high vacuum sputter coater (Leica, Wetzlar, Germany), a 100 nm gold layer on glass substrates previously cut with a diamond tip. Polyester fabric substrates (Servei Estació, Barcelona, Spain) were cut and blown with nitrogen before use.

### 2.2. Preparation of the Primer Coatings for Their Physicochemical Characterization

Primer coatings were synthesized in 15 mL glass vials by dissolving benzene-1,2-diol (10 mmol) and hexamethylenediamine (HMDA, 10 mmol) in carbonate buffer (6 mL, pH = 9.1). The substrates (glass, gold or silicon) were placed in the vials together with the reagents. Then, the carbonate buffer was added under homogenous magnetic stirring. The vials were covered with pierced Parafilm^®^ (Labbox) in order to allow the entrance of oxygen to the reaction mixture. Finally, the samples were washed with Milli-Q^®^ water, dried with air flow and stored in a desiccator under vacuum.

### 2.3. Functionalization of the Primer Coatings with Hydrophobizing Agents

Primer-coated glass substrates were placed in 15 mL glass vials together with 6 mg of the corresponding hydrophobizing agent (1-hexadecanamine or stearoyl chloride). Then, 6 mL of hexane were added, the vial closed and kept under magnetic stirring for 24 h. Finally, the substrates were washed with hexane, dried with air flow and stored in a desiccator under vacuum conditions.

Variations of the contact angle values due to the presence of adsorbed solvent molecules were discarded by preparing blank samples of the coatings, following the above protocol but without addition of hydrophobizing agents.

### 2.4. Material Coating and Functionalization under Optimized Conditions

The substrates (fabric, silicon and gold) were placed horizontally in glass petri dishes for the deposition and formation of the primer coatings following the process described in Section 2.2. The resulting primer-coated substrates were then incubated for 24 h in glass petri dishes containing 1 mg/mL of hydrophobizing agent (1-hexadecanamine or stearoyl chloride) in hexane. Finally, the substrates were washed with hexane, dried with air flow and stored in a desiccator under vacuum conditions.

### 2.5. Contact Angle

Static contact angle measurements were performed with an EasyDrop contact angle meter (KRÜSS GmbH, Hamburg, Germany) using 15 μL water droplets. Each substrate was measured at three different points for obtaining an average of the whole surface. The measurements were performed approximately one minute after the droplet deposition.

### 2.6. Chemical Characterization

Ultraviolet–visible (UV–Vis) spectra have been acquired on glass substrates using a Cary 4000 Spectrometer (Agilent Technologies, Santa Clara, CA, USA). Surface FT-IR experiments have been performed with the Hyperion 2000 FT-IR microscope (Bruker Optik GmbH, Ettlingen, Germany) in reflection mode equipped with a nitrogen cooled mercury–cadmium–telluride (MCT) detector (InfraRed Associates, Inc., Stuart, FL, USA) using a 15× reflection objective, a gold mirror as reference and scanning for 30 min with a resolution of 4 cm^−1^. X-ray photoelectron spectroscopy (XPS) measurements were performed with a Phoibos 150 analyser (SPECS EAS10P GmbH, Berlin, Germany) in ultra-high vacuum conditions (based pressure 10^−10^ mbar, residual pressure around 10^−7^ mbar). Monochromatic Al Kα line was used as X-ray source (1486.6 eV and 300 W). The electron energy analyser was operated with pass energy of 50 eV. The hemispherical analyser was located perpendicular to the sample surface. The data was collected every eV with a dwell time of 0.5 s. A flood gun of electrons, with energy lower than 20 eV, was used to compensate the charge. The primer coatings were deposited on silicon substrates during three different times: 12, 24 and 48 h. All the data was treated with CasaXPS version 2.3.17PR1.1 (Casa Software LTD, Teignmouth, UK) and OriginPro version 8.0988 (OriginLab Corporation, Northampton, MA, USA) software.

### 2.7. Morphological Characterization of the Coatings

Measurements of the coating thickness were obtained from a Stylus Profilometer (D-500, KLA Tencor, Milpitas, CA, USA), with a vertical range of 1200 μm; 0.38 Å and 100 nm as vertical and lateral resolution, respectively. The measurements were performed with stylus force in the range of 1–5 mg. The primer coatings were deposited on glass substrates with a mask in the middle for the formation of a step suitable for the thickness measurement.

Images of treated fabric were obtained from scanning electron microscopy (SEM) (FEI Quanta 650 FEG, Thermo Fisher Scientific, Eindhoven, The Netherlands) in secondary electron mode with a beam voltage between 15 and 20 kV. Samples were coated with 15 nm of platinum by sputter coater (Leica).

### 2.8. Statistical Analysis

The statistical analysis of the contact angle measurements was carried out by performing three replicates per sample. The obtained results per sample were used for calculating the standard deviation using the *n*–1 method with Excel version 2010 (Microsoft Office, Redmond, WA, USA) software.

## 3. Results and Discussion

### 3.1. Generation of the Primer Coating

**Figure 2:**
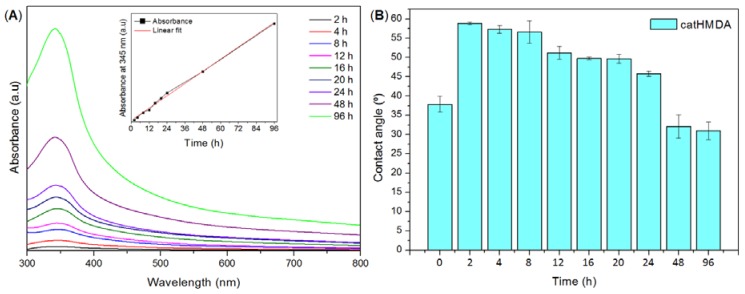
Study of the catHMDA growth with time. (**A**) Ultraviolet–visible (UV–Vis) spectra of catHMDA on glass vs. reaction time. Inset shows the linear trend of the maximum of absorbance at 345 nm as a function of the reaction time. (**B**) Static contact angle measurements of catHMDA-coated glass as a function of the reaction time. Data is shown as mean ± standard deviation. a.u.: Arbitrary units.

In a first attempt to generate a functionalizable primer coating by cross-linking of pyrocatechol with a nitrogenated species, three different reagents were tested for copolymerization in basic media: NH_3_ aqueous solution (25% *w*/*w*), *p*-phenylenediamine and HMDA. Reactions using NH_3_ or *p*-phenylenediamine yielded no coating on glass substrates. Despite the fact that an evident darkening of the solutions took place with time during these reactions, no material remained on the glass substrates after cleaning with water, as could be clearly observed with the naked eye, but also with UV–Vis spectroscopy. Finally, contact angle measurements were also performed on these samples, but values did not significantly vary from those of the untreated glass. Thus, the pyrocatechol polymerization products obtained with NH_3_ or *p*-phenylenediamine did not seem to be able to generate a stable coating on this substrate. In the case of HMDA, a brownish coating remained on glass after thoroughly cleaning with water. Ultraviolet–visible spectroscopy showed a band around 345 nm, which was assigned to the polymerization product between pyrocatechol and HMDA (catHMDA). This technique was also used for following coating growth with time (Figure 2A). Surprisingly, no maximum was reached even after four days of reaction, and the deposition of material seemed to follow a linear trend, without appreciable shift of the position of the band (inset in Figure 2A).

**Figure 3:**
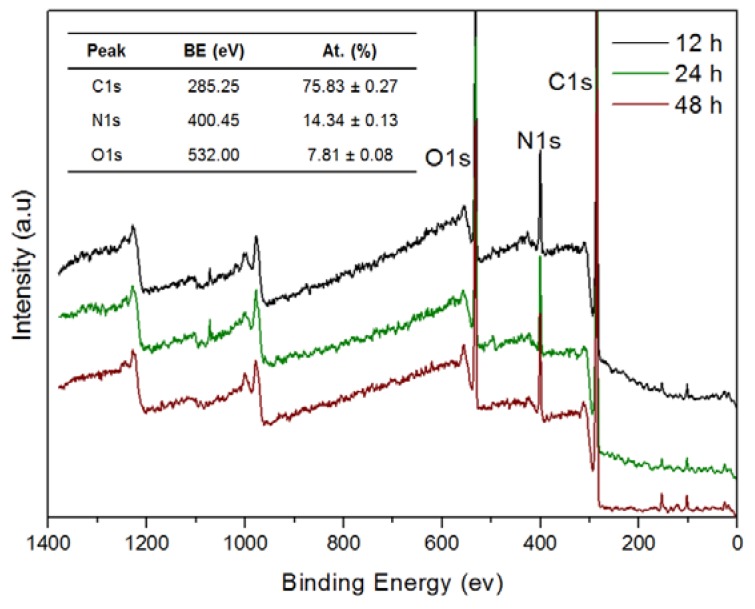
X-ray photoelectron spectroscopy (XPS) spectra of the primer coatings deposited on silicon substrate at different reaction times. The inset table shows the binding energy (BE) and the atomic concentration (At.) of C, N and O. a.u.: Arbitrary units.

This observation is also supported by profilometry measurements, showing that the measured thickness of samples at 12, 24, 48 and 96 h also follows a nearly linear trend, being of 55 ± 8, 95 ± 5, 160 ± 10 and 386 ± 7 nm, respectively. Thus, it seems that under the reaction conditions used in these experiments, catHMDA grows on the glass substrates at an essentially constant rate during at least the first four days. A feasible explanation for this phenomenon is that, since the cross-linking reaction needs the prior oxidation of the catechol to the corresponding quinone and, in turn, this step needs the presence of oxygen, the rationed entrance of this last to the reaction mixture makes the process to run gradually. However, static contact angle measurements of the same samples showed that a significant decrease of this value takes place with time, parallel to the growth of the material (Figure 2B). A first quick increase of the contact angle in relation to the naked surface can be observed after 2 h of reaction. From here, the surface becomes more hydrophilic as the reaction time increases. These results suggest that the outer surface of the coating exposes more hydrophilic moieties as the reaction progresses. Nevertheless, and unlike layer thickness, no linearity was observed for this parameter. In fact, three different stages could be clearly differentiated regarding the variation of contact angle with time: between 2 and 8 h, when the highest contact angles around 57° are obtained; from 12 to 24 h, where a slight decrease down to around 50° may be appreciated; and between 48 and 96 h, when a dramatic drop of this parameter down to around 30° takes place, being even lower than that of the blank surface. It is worth mentioning that these values were reproducible after several weeks, demonstrating the robustness of the coating. Overall, results suggest that although the generation of the coating seems to take place gradually as mentioned above, its chemical composition should be varying with time, considering the availability of reactants in the medium (starting ratio of catechol:HMDA 1:1.5). As the catechol monomer is depleted, excess amine might favor the formation of more hydrophilic layers with free amine chain ends, which would explain the observed drop in contact angle values. In order to shed some light on this issue, XPS was used for the analysis of atomic chemical bonding of the primer coatings on silicon substrates at three different times: 12, 24 and 48 h. In all the cases, the C1s, N1s and O1s peaks yielded a very similar spectrum as expected and appeared in a closely position with a concentration of 75.83 ± 0.27%, 14.34 ± 0.13% and 7.81 ± 0.08%, respectively (Figure 3).

**Figure 4:**
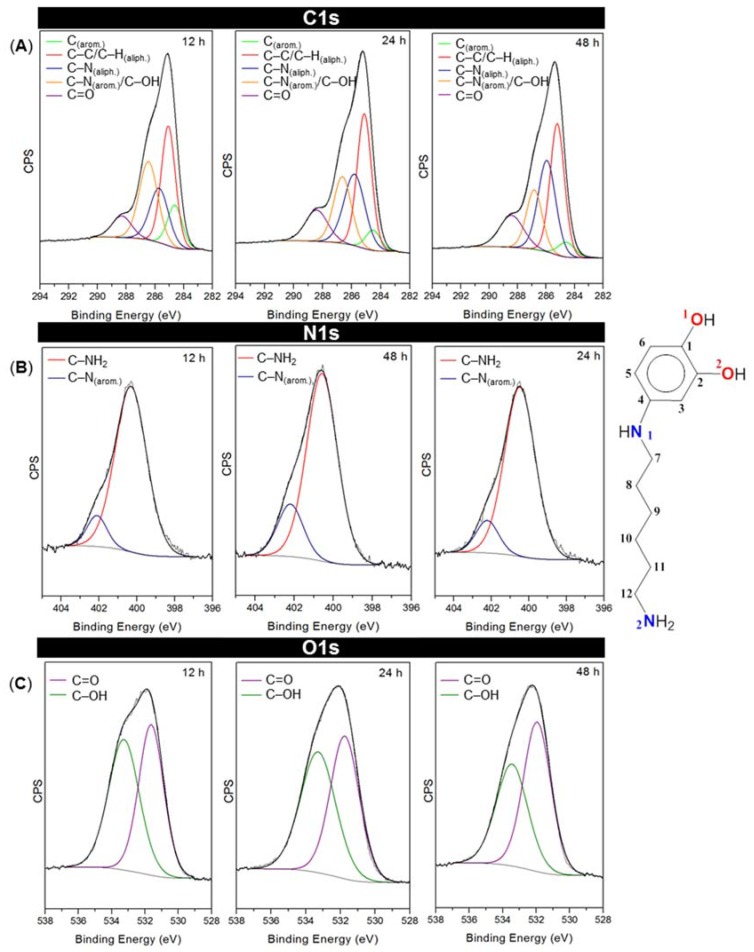
Curve-fitting results for C1s, N1s and O1s high-resolution XPS spectra at 12, 24 and 48 h. CPS: Counts per second. The scheme on the right represents the different kinds of chemical bonds in catHMDA. The atoms are arbitrary numbered for the XPS peak assignment.

To study in detail the bonding environment of the coating deposited on SiO_2_, high-resolution XPS curve-fitting was performed. Figure 4 shows the XPS spectra with the fitting results for each peak of the primer coatings at the studied times. Table 1 shows the corresponding assignments for the resulting components. The energies were in agreement with previously reported values for similar systems [43,44,45,46,47].

Figure 4A shows the C1s spectra for each time. Five peaks have been properly fitted, which would correspond to five different chemical environments in the primer coating. They confirm the presence of catechol coexisting with its oxidized quinone state as can be noted by the C–OH signals at 286.45, 286.64 and 286.83 eV, and the C=O signals at 288.31, 288.42 and 288.43 eV for 12, 24 and 48 h reaction times, respectively. The O1s spectra (Figure 4C) also support the coexistence of these two species. In addition, it can be observed that the peaks corresponding to the reduced (catechol) state (both at C1s and O1s spectra) decrease with time, whereas those assigned to quinonic species increase, which would indicate an over-oxidation of catechol moieties at progressively longer reaction times. Regarding N1s spectra (Figure 4B), an increase of both aliphatic and aromatic amine-related components can be observed. C–N aromatic contributions could be assigned to the amino groups that are directly bonded to the catechol rings and are supposed to be involved in the cross-linking of the material, whereas C–NH aliphatic would be indicating the presence of unreacted amine tail ends. Although both signals rise with time, it can be noted that the one corresponding to the unreacted (aliphatic) amine tails undergoes a more significant increase.

This would be in agreement with our previous statement about the drop in the contact angle values for long reaction times, since free amine-rich top layers would confer hydrophilicity to the primer coating surface.

**Figure 5:**
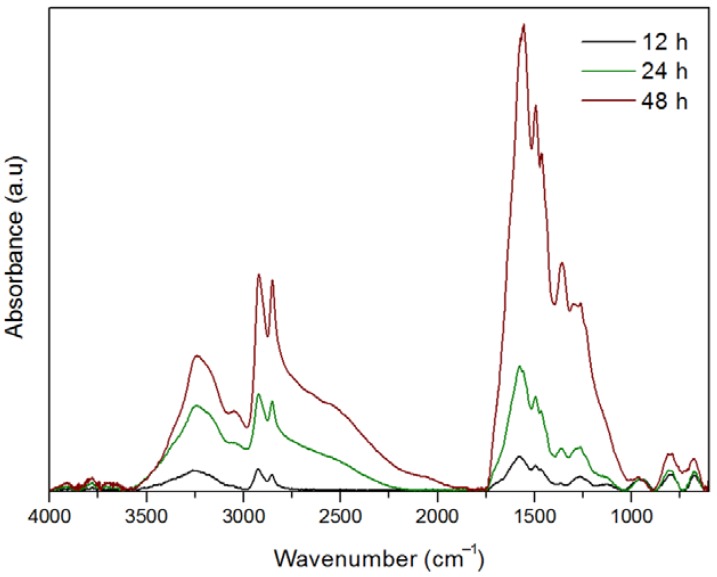
Fourier-transform infrared spectroscopy (FT-IR) spectra of the primer coatings after 12, 24 and 48 h of reaction. a.u.: Arbitrary units.

Characterization of the primer coatings obtained at 12, 24 and 48 h by means of FT-IR also shows, on the one hand, an increase in the thickness of the coating with time, as can be noted by the increase in absorbance of the corresponding spectra (Figure 5).

On the other hand, FT-IR clearly shows that both HMDA and catecholic/quinonic species are present in the coating. The broad band around 3240 cm^−1^ and its shoulder at higher wavenumbers around 3400 cm^−1^ may be assigned to the NH_2_ and O–H vibrations from catechol and amine moieties, respectively. The bands observed in all the spectra at 3050 cm^−1^, and between 1560 and 1580 cm^−1^ can be respectively assigned to C=C–H and C=C vibrations from the catecholic/quinonic rings, whereas peaks around 2853 and 2924 cm^−1^ would correspond to the asymmetric and symmetric stretching vibrations of the methylene C–H bonds from the alkyl chain of the HMDA. All spectra present shoulders of the intense bands around 1575 cm^−1^, and between 1620 and 1710 cm^−1^. Although they are not perfectly resolved, it is feasible to assign them to C=O quinonic groups, that otherwise have also been observed, as described above, by XPS. Finally, the peak at 1264 cm^−1^ observed in all the spectra could be assigned to a secondary amine bridging an alkyl and an aromatic ring. Since these measurements are a read of the whole material (unlike XPS, as it is not a superficial technique) and, in addition, the resolution of the bands is not optimal mainly for shorter reaction times, it is complicated by only means of FT-IR to establish an evolution of the functional groups with time. Nevertheless, these findings along with the XPS, UV–Vis and profilometry data would suggest that the primer coating consists in a cross-linkage of catechol/quinone rings through HDMA that gradually increases its thickness with time in an almost linear trend. The longer the time of reaction, the higher the concentration of quinones, and unreacted tail-end amines can be found in the surface of the coating. Thus, its reactivity towards a given functionalizing agent should be considered in function of the time of its generation.

### 3.2. Functionalization of the Primer Coating

**Figure 6:**
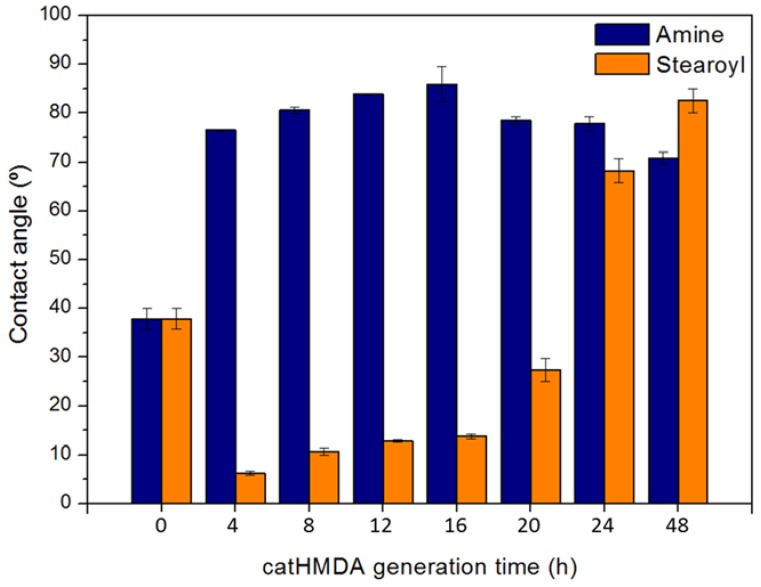
Contact angles of catHMDA generated at different times (4–48 h), after incubation with 1-hexadecanamine (blue) and stearoyl chloride (orange). Both incubations are carried out for 24 h in hexane. Data is shown as mean ± standard deviation.

Considering the presence of quinones and primary amines in the surface of the catHMDA coating, its reactivity towards a nucleophilic and an electrophilic attack as functionalization strategies was explored and evaluated. For this, an amine and an acyl chloride, both bearing alkyl chains, were used as functionalization agents, since their successful attachment may be easily tested by measuring the resulting contact angle. Thus, catHMDA coatings were generated at different times and later incubated with the functionalizing agents (Figure 6). As mentioned above, the reactivity of the primer coating would be expected to change with reaction time, concomitantly with changes in its chemical composition.

In Figure 6, different trends for the functionalizing agents can be observed. Although both maxima achieve similar values around 85°, the amine nucleophilic attack seems to be more successful on the catHMDA obtained after 16 h (catHMDA-16 h), whereas the electrophilic attack of the acyl chloride seems to be optimum for the catHMDA coating generated for 48 h (catHMDA-48 h).

This trend would support the primer coating characterization results: on one hand, the longer time for the generation of catHMDA, the larger number of primary amines can be found in the surface, thus increasing the probability of being attacked by an electrophilic reagent during the subsequent functionalization incubation. On the other hand, it has also been observed that the amount of quinones, liable to later reacting with nucleophiles, increases with the generation time of catHMDA. Nonetheless, HMDA can react with these quinones during the generation of the primer coating, thus blocking reactive positions towards other nucleophiles in the later functionalization process. In fact, this could be the reason why a larger number of tail-end amines can be found as an overlayer on the coating for longer reaction times by means of XPS (Table 1, N1s). Considering these observations, it seems feasible that at a certain point the reactivity of catHMDA towards nucleophiles would decrease, with a concomitant increase in reactivity towards electrophiles. This point of inflection can be observed in Figure 6 approximately after 16 h. The extremely low contact angles obtained up to 20 h for the incubation with stearoyl chloride, being even lower than the uncoated glass substrate, could be due to a chemical modification of the coating surface during this process. An increase in the number of primary amines on the surface for longer generation times, besides increasing the reactivity towards an acyl chloride, could be buffering such side effects. Nevertheless, further studies would be needed to shed light on this issue.

**Figure 7:**
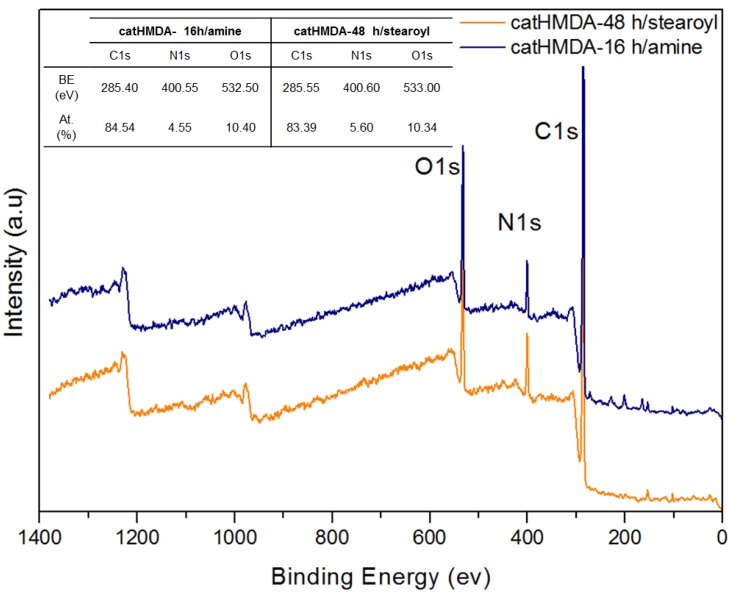
XPS spectra of the functionalized primer coatings deposited on a silicon substrate. The inset table shows their chemical composition. a.u.: Arbitrary units.

In order to confirm the attachment of the functionalizing agents, as well as to determine the mechanisms involved in the corresponding processes, the functionalized coatings showing optimal contact angles (catHMDA-16 h/amine and catHMDA-48 h/stearoyl) were analysed by XPS. Figure 7 shows the wide scan XPS spectra and the compositional analysis, where an increase of the atomic percentage of carbon can be noted, reaching values higher than 80%, which would be compatible with the attachment of the bulky alkyl chains to the surface. Additionally, the presence of N and O was also confirmed.

**Figure 8:**
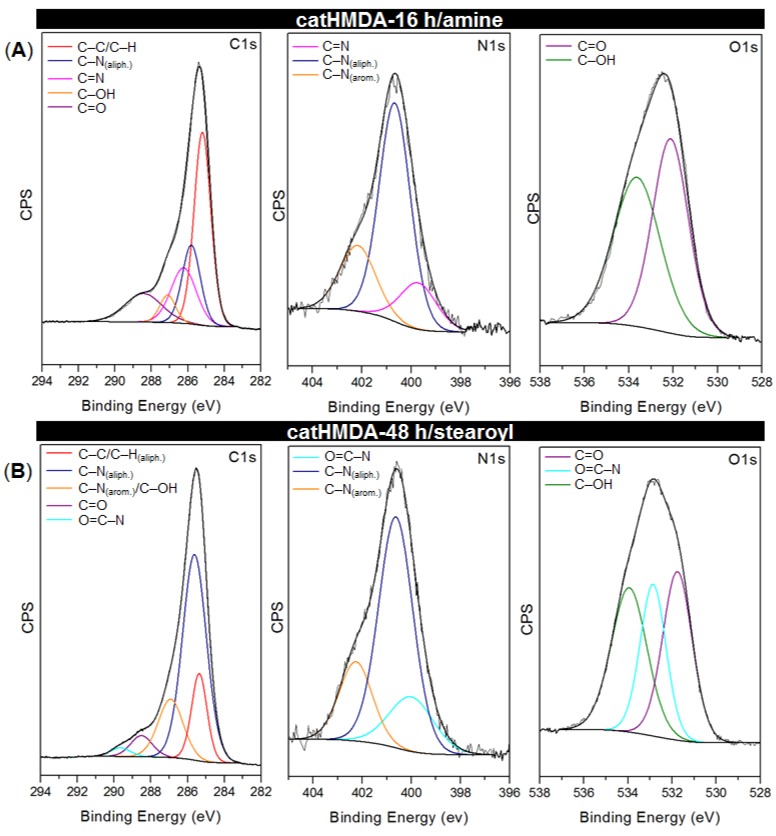
Curve-fitting results corresponding to C1s, N1s and O1s high-resolution XPS spectra for (**A**) catHMDA-16 h/amine and (**B**) catHMDA-48 h/stearoyl.

The core-level spectra C1s, N1s and O1s for both functionalized coatings are presented in Figure 8, and relevant peak data and assignments listed in Table 2. The energies are in agreement with reported values for similar chemical bonding [43,48,49]. In both systems, besides the signals coming from the underlying catHMDA primer coating, new signals which could be assigned to the new established bonds after the incubation process can be observed. In the case of catHMDA-16 h/amine, the C1s spectrum (Figure 8A) shows a component at 286.23 eV that can be assigned to the formation of an imine bond (C=N), also confirmed in the N1s spectrum with a signal at 399.73 eV.

**Figure 9:**
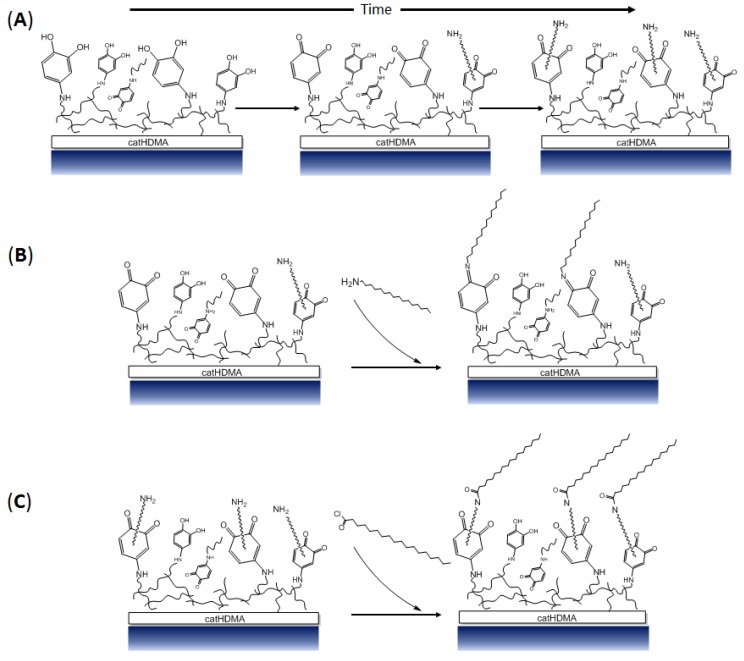
Chemical structure of the catHMDA coating and its functionalization. (**A**) Tentative schematic representation of the primer coating (catHDMA) and its evolution in function of the reaction time, where an increase in the amount of quinones and non-reacted amine tail ends can be observed. (**B**) Proposed schematic mechanism of the attachment of 1-hexadecanamine to catHDMA-16 h (the optimal generation time of the primer coating for its functionalization with this reagent is 16 h) (**C**) Proposed schematic mechanism of the attachment of stearoyl chloride to catHDMA-48 h (the optimal generation time of the primer coating for its functionalization with this reagent is 48 h).

This would hint at covalent bonds being formed by nucleophilic attacks of amines on the carbonyl groups of the quinone rings (Figure 9B), which seem to be present in large amounts for intermediate reaction times (Figure 9A). With regard to Michael-type additions, they cannot be completely ruled out, since signals at 400.67 and 402.17 eV, corresponding to secondary amines bridging aromatic and aliphatic carbons, are also observed in this spectrum. Although cross-linking of catechol rings through HMDA is the main origin of such signals, some contribution coming from the attachment of 1-hexadecanamine to the aromatic ring by means of a 1,4-addition is also feasible in this context. In the case of catHMDA-48 h/stearoyl (Figure 8B), one of the fitted component in C1s peak with highest binding energy (289.67 eV) can be attributed to an amidic carbon atom (N–C=O), thus confirming the condensation between free amines in the catHMDA and stearoyl chloride (Figure 9C). The presence of the amide bond is also confirmed by the 532.86 eV peak in the O1s high-resolution spectrum.

Chemical characterization by means of FT-IR has also been performed after the functionalization processes, but no significant peaks coming from these overlayers have been detected due to the considerable larger thickness of the underlying catHMDA coating, which absorbs most of the FT-IR signal. Nevertheless, contact angle and XPS measurements have been enough for confirming the successful functionalization of the catHMDA primer coating with 1-hexadecanamine and stearoyl chloride.

**Figure 10:**
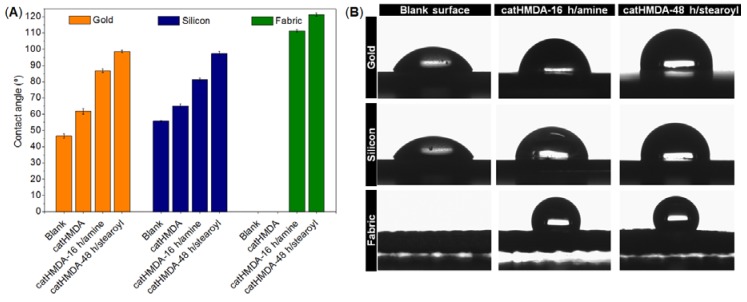
Wettability study after functionalization of catHDMA. (**A**) Contact angles of water on gold, silicon and fabric substrates (blank/uncoated, coated with catHMDA, and coated + functionalized with 1-hexadecanamine and stearoyl chloride). Data is shown as mean ± standard deviation. (**B**) Images of water droplets on the three substrates before and after functionalization with catHMDA-16 h/amine and catHMDA-48 h/stearoyl.

Finally, three different substrates (gold, silicon and fabric) were rendered hydrophobic in the optimal conditions for the generation of the primer coatings and their further functionalization. Contact angle values at the different stages of this treatment are shown in Figure 10.

**Figure 11:**
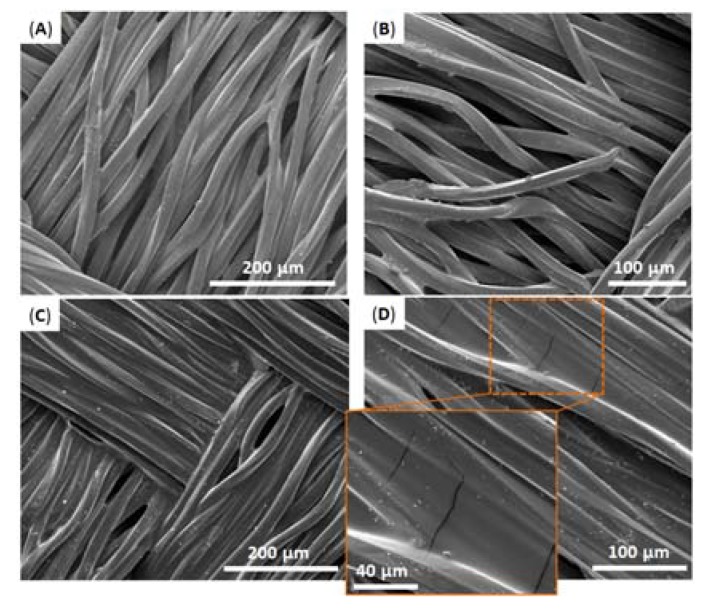
Scanning electron microscopy (SEM) images of fabric samples (**A**,**B**) before and (**C**,**D**) after being coated with catHMDA-16 h/amine. Inset in (**D**) shows zoom area of the coated polyester fabric fibers, where small cracks can be observed on its surface.

As can be seen in Figure 10A, in all the cases catHMDA-48 h/stearoyl leads to the highest contact angle values, approximately 10° above those obtained with catHMDA-16 h/amine. Interestingly, it can be noted that for substrates having similar roughness, like gold and silicon, the contact angle values are almost equal for the same treatment. This could be evidence for the universality of this coating, which would provide the same functionalization capacities independently of the chemical nature of the treated surface. The greater roughness of the fabric substrate causes a significant increase in the final contact angles, due to the structuration of the surface. The morphology of this last sample coated with catHMDA-16 h/amine has been analyzed by SEM. As shown in Figure 11, almost no differences in appearance can be seen between the untreated (Figure 11A,B) and the treated fabric (Figure 11C,D), where the presence of a coating is hinted at by small cracks across its surface, as observed at higher magnification (Figure 11D and zoom inset). Thus, even after 16 h of generation of the coating, the submicron thickness of the coating does not significantly modify the morphology of the treated textile, which is important for preserving its mechanical and breathability properties.

## 4. Conclusions

A functionalizable polydopamine-like coating (catHMDA) was successfully obtained by cross-linking polymerization of pyrocatechol with HMDA under oxidizing conditions. Regarding the growth of the coating, UV–Vis spectroscopy and profilometry measurements show that the thickness of catHMDA on flat surfaces follows an almost linear trend with time under the reaction conditions. The XPS measurements suggest that its superficial chemical composition also changes with time, thus opening the possibility of tuning its reactivity in function of this parameter. As a proof-of-concept, different substrates were coated with catHMDA and later functionalized with 1-hexadecanamine and stearoyl chloride, demonstrating the versatility of this platform for its functionalization following both nucleophilic and electrophilic attack approaches. The bulky alkyl chain in both species successfully conferred robust hydrophobicity to four different surfaces (glass, gold, silicon and fabric). These results demonstrate the potential of catHDMA as a universal coating that can act as a flexible platform for further functionalization with, for example, biomolecules of interest in biomaterials science, amongst others. However, future work should be directed in order to explore the degradability of these coatings. In addition, the use of more superficial techniques could be of interest for shedding more light on the chemical composition of the top atomic layers of these systems.

## Figures and Tables

**Table 1:** Curve-fitting assignments for C1s, N1s and O1s peaks at 12, 24 and 48 h.

Peak	Time ^1^ (h)	Atom	Atomic Bond	BE (eV)	At. (%)
**C1s**	12	C3,5,6	C_(arom.)_	284.61	12.06
		C8,9,10,11	C–C/C–H_(aliph.)_	285.07	32.52
		C7,12	C–N_(aliph.)_	285.71	19.48
		C4/C1,2	C–N_(arom.)_/C–OH	286.45	27.87
		C1,2	C=O	288.31	8.07
	24	C3,5,6	C_(arom.)_	284.53	5.34
		C8,9,10,11	C–C/C–H_(aliph.)_	285.13	34.64
		C7,12	C–N_(aliph.)_	285.81	26.61
		C4/C1,2	C–N(_arom._)/C–OH	286.64	21.04
		C1,2	C=O	288.42	12.37
	48	C3,5,6	C_(arom.)_	284.57	4.52
		C8,9,10,11	C–C/C–H_(aliph.)_	285.22	36.52
		C7,12	C–N_(aliph.)_	285.94	29.64
		C4/C1,2	C–N_(arom.)_/C–OH	286.83	15.27
		C1,2	C=O	288.43	14.05
**N1s**	12	N1	C–NH_2_	399.87	81.26
		N2	C–N_(arom.)_	401.11	10.51
	24	N1	C–NH_2_	399.08	82.14
		N2	C–N_(arom.)_	401.65	11.26
	48	N1	C–NH_2_	399.87	88.49
		N2	C–N_(arom.)_	401.78	11.51
**O1s**	12	O1,2	C=O	531.63	48.39
		O1,2	C–OH	533.27	51.61
	24	O1,2	C=O	531.75	49.41
		O1,2	C–OH	533.29	50.59
	48	O1,2	C=O	531.95	56.08
		O1,2	C–OH	533.45	43.92

^1^ catHMDA generation time. aliph.: Aliphatic; arom.: Aromatic; At.: Atomic concentration; BE: Binding energy.

**Table 2:** Curve-fitting assignments corresponding to C1s, N1s and O1s peaks for catHMDA-16 h/amine and catHMDA-48 h/stearoyl.

catHMDA ^1^	Peak	Atomic Bond	BE (eV)	At. (%)
**Amine**	C1s	C–C/C–H_(aliph.)_	285.20	42.92
		C–N_(aliph.)_	285.81	18.93
		C=N	286.23	18.51
		C–OH	287.11	5.73
		C=O	288.43	13.91
	N1s	C=N	399.73	15.62
		C–N_(aliph.)_	400.67	62.72
		C–N_(arom.)_	402.17	21.66
	O1s	C=O	532.12	49.84
		C–OH	533.64	50.16
**Stearoyl**	C1s	C–C/C–H_(aliph.)_	285.37	16.29
		C–N_(aliph.)_	285.63	58.94
		C–N_(arom.)_/C–OH	286.93	17.20
		C=O	288.51	5.56
		O=C–N	289.67	2.01
	N1s	O=C–N	399.98	18.51
		C–N_(aliph.)_	400.63	61.30
		C–N_(arom.)_	402.26	20.19
	O1s	C=O	531.76	35.53
		O=C–N	532.86	27.88
		C–OH	533.95	36.59

^1^ Optimized catHMDA for each functionalization. aliph.: Aliphatic; arom.: Aromatic; At.: Atomic concentration; BE: Binding energy.
